# Study on the release pattern of Zn in soil of ionic rare earth mining areas under different leaching conditions

**DOI:** 10.1371/journal.pone.0338566

**Published:** 2025-12-15

**Authors:** Zhongqun Guo, Qiangqiang Liu, Feiyue Luo, Shaojun Xie, Tianhua Zhou

**Affiliations:** 1 School of Civil Engineering and Surveying and Mapping Engineering, Jiangxi University of Science and Technology, Ganzhou, China; 2 Jiangxi Provincial Key Laboratory of Water Ecological Conservation in Headwater Regions, Ganzhou, China; University of Tehran, IRAN, ISLAMIC REPUBLIC OF

## Abstract

The acidic leachate injected during the mining process of ion-type rare earth ores can damage the environmental characteristics of the soil, thereby triggering the activation and release of associated heavy metals. Severe Zn contamination has been found in the environment of ion-type rare earth mining areas, but the activation and release of Zn in the soil during the leaching process have not been fully understood. This study investigated the activation and release patterns and mechanisms of Zn in soil under different leaching agents ((NH_4_)_2_SO_4_, MgSO_4_, Al_2_(SO_4_)_3_) and varying concentrations of Al_2_(SO_4_)_3_ (1%, 3%, 5%, 7%) using a simulated leaching experimental system. The results show that the activation and release patterns of Zn in the soil vary significantly under the influence of the three leaching agents. During the entire leaching cycle, the peak Zn concentration in the leachate was highest under MgSO_4_ leaching, while the residual Zn content in the soil under Al_2_(SO_4_)_3_ leaching approached the high-risk environmental threshold. The high-concentration systems (5%, 7%) of Al_2_(SO_4_)_3_ significantly enhanced the activation and release efficiency of Zn in the soil compared to the low-concentration systems (1%, 3%) of Al_2_(SO_4_)_3_. (NH_4_)_2_SO_4_ mainly promotes the activation and release of Zn through ion exchange between NH_4_^+^ and Zn^2+^ and the acidification effect; Al_2_(SO_4_)_3_, on the other hand, dominates the activation and release of Zn by providing a strongly acidic environment and dissolving and damaging the mineral lattice; while MgSO_4_ not only exchanges ions between Mg^2+^ and Zn^2+^, but also alters the soil colloidal structure, facilitating Zn activation and release. The promoting effects of the three leaching agents on the transformation of Zn in soil follow the order of Al_2_(SO_4_)_3_> (NH_4_)_2_SO_4_ > MgSO_4_, with the environmental risk assessment index (RAC) being highest after Al_2_(SO_4_)_3_ leaching, indicating the greatest potential environmental risk. Compared to the other three concentrations (1%, 5%, 7%) of Al₂(SO_4_)_3_, the 3% concentration of Al_2_(SO_4_)_3_ had the most significant promoting effect on the transformation of Zn in soil. This study provides a theoretical basis for optimizing the green mining process of ion-type rare earth ores and preventing heavy metal pollution, and offers scientific support for revealing pollution mechanisms and formulating remediation and risk assessment strategies.

## 1. Introduction

Ion-type rare earth resources contain rich medium and heavy rare earth elements, which are highly valuable, limited in reserves, and possess high technological added value. These resources are considered strategic minerals with global attention, primarily distributed in the southern regions of China [1]. In such deposits, rare earth elements are adsorbed onto clay minerals in the form of hydrated cations or hydroxy-hydrated cations, and are typically exploited using salt leaching agents. In industrial production, processes such as pond leaching, heap leaching, and in-situ leaching have been successively employed, with in-situ leaching widely recommended [2,3]. In this method, drilling is performed on-site at the mine, and leaching agents are injected. Through chemical exchange reactions, rare earth ions are desorbed, forming a leachate to achieve the enrichment of rare earth elements [4].

However, during the in-situ leaching of ion-type rare earth ores, a large amount of acidic leaching solution (pH 2–5) needs to be injected into the soil. While displacing rare earth cations, it also displaces some highly oxidizing heavy metal cations, which can damage the internal structure of the soil, cause an imbalance in the soil buffering system, and promote the activation and release of heavy metals in the soil [5]. This results in the release of heavy metals into the rare earth leachate, leading to changes in their chemical forms [6]. The geological conditions of ion-type rare earth mines are typically complex, with issues such as leakage from impermeable layers and imperfect liquid collection systems in some areas. The heavy metals in the rare earth leachate can easily migrate into the mine’s water bodies through underground leakage and surface runoff, and even accumulate in the soil of the mining area, leading to severe heavy metal contamination issues [7].

Zinc is an essential trace element for cellular metabolism, but excessive Zn in the environment can not only be toxic to plants but also pose a threat to human health through the food chain. Many studies have found that modern mining operations can lead to severe Zn pollution [8]. Li et al. [9] found that the large amounts of ammonium ions (NH^4+^) remaining in the mining area after leaching can alter the chemical forms and mobility of heavy metals such as Zn, Cu, and Cd, leading to ammonium nitrogen pollution and heavy metal contamination in rare earth mining areas. Tan et al. [10] found that ammonium sulfate leaching promoted the precipitation of heavy metals, with the total contents of Cu, Zn, and Cr in the soil decreasing by 18.34%, 10.96%, and 26.00%, respectively, with Cu and Zn mainly leached in their weak acid forms. Huang et al. [11] studied an abandoned ion-type rare earth mining area in Chongzuo, Guangxi, and found that the Zn content in the surface soil ranged from 57 to 121 mg/kg, and from 50 to 139 mg/kg in the deeper soil, both of which exceeded the national background values for soil elements. Zhang et al. [5] conducted a study on an ion-type rare earth mining area in southern Jiangxi, and found that the average concentrations of heavy metals Cd, Hg, As, Pb, and Zn in the area were 0.3, 1.0, 32.6, 135.9, and 113.2 mg/kg, respectively, all of which exceeded the background values for soil environment in Jiangxi Province. Therefore, Zn pollution caused by leaching is a common issue in ion-type rare earth mining, but existing research mainly focuses on the Zn pollution in water and soil of mining areas after leaching with (NH_4_)_2_SO_4_ and MgSO_4_. There is still insufficient research on the activation and release patterns of Zn in mining area soils during leaching and the Zn pollution of soils after leaching with the novel leaching agent Al_2_(SO_4_)_3_.

The environmental impact of heavy metals in soil largely depends on their chemical forms [12]. The acid-extractable fraction of heavy metals is easily released in weak acidic environments, posing the greatest environmental hazard, whereas the residue fraction is almost impossible to release under natural conditions, resulting in a very low environmental risk [13]. The activated release of Zn in ion-type rare earth mining area soils is, in fact, the result of leaching agents inducing the chemical form transformation of Zn in the soil. Leaching agents, as the sole exogenous factor inducing heavy metal pollution in ion-type rare earth mines, affect the chemical form transformation of heavy metals in soils differently due to their unique chemical properties (such as pH, complexing ability, redox potential, etc.) [14]. Currently, studies on the evolution of the chemical forms of Zn in soils of ion-type rare earth mining areas under different leaching conditions are still relatively scarce.

This study focuses on the Zn content in the soil of an ion-type rare earth mining area in southern China. A custom-designed column leaching test device was used to simulate leaching processes with different leaching agents ((NH_4_)_2_SO_4_, MgSO_4_, Al_2_(SO_4_)_3_) and varying Al_2_(SO_4_)_3_ concentrations (1%, 3%, 5%, 7%). During the leaching period (0–7 days), the Zn content in the rare earth leachate and soil was monitored to reveal the activation and release patterns of Zn in soil under the influence of different leaching agents and concentrations. Finally, the BCR sequential extraction method was used to determine the proportions of four different chemical forms of Zn in the soil under varying leaching conditions. The effects of different leaching agents and their concentrations on the chemical forms of Zn in soil were analyzed, and the activation and release mechanism of Zn in the soil of ion-type rare earth mining areas was explored from the perspective of chemical forms. Additionally, an environmental risk assessment of Zn in the soil before and after leaching was conducted. The aim is to provide valuable references for the prevention and control of Zn pollution in ion-type rare earth mining areas and to offer theoretical support for optimizing green mining processes and heavy metal pollution control in ion-type rare earth mining.

## 2. Materials and methods

### 2.1. Experimental materials

#### 2.1.1. Sample collection.

**Fig 1:**
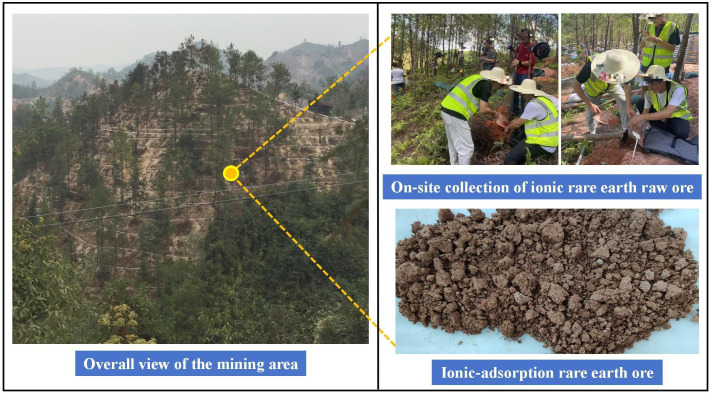
Schematic of the mining area overview and the on-site sampling process.

The soil samples of the raw ore were collected from an ionic rare earth mining area in Fujian Province, as shown in Fig 1. After sampling, the raw ore soil samples were placed in sealed bags and transported to the laboratory, where the moisture content was measured using the drying method and was found to be 15.24%. After removing debris, stones, and other impurities, the soil samples were spread out in a cool indoor area to air dry naturally. After air drying, the samples were crushed and stored in sealed bags for later use. To avoid external metal contamination, non-metallic tools were used throughout the sampling and processing of the soil samples.

#### 2.1.2. Mineral composition analysis.

(1) Soil chemical composition analysis

The chemical composition of the treated soil was analyzed using X-ray fluorescence spectroscopy (XRF), and the results are shown in Table 1. As shown in Table 1, the main chemical components of the rare earth ore are SiO_2_ (68.201%), Al_2_O_3_ (22.069%), and Y_2_O_3_ (0.003%). This indicates that the soil samples from the study area are primarily composed of aluminosilicate minerals and belong to the Y-rich heavy rare earth soil type.

**Table 1:** Chemical composition of soil samples.

Chemical composition	SiO_2_	MgO	Al_2_O_3_	K_2_O	Fe_2_O_3_	CaO
Content (%)	68.201	0.161	22.069	1.453	3.916	0.009
Chemical composition	MnO	Rb_2_O	PbO	ZnO	Y_2_O_3_	
Content (%)	0.046	0.014	0.015	0.007	0.001

(2) Analysis of soil mineralogical composition

**Fig 2:**
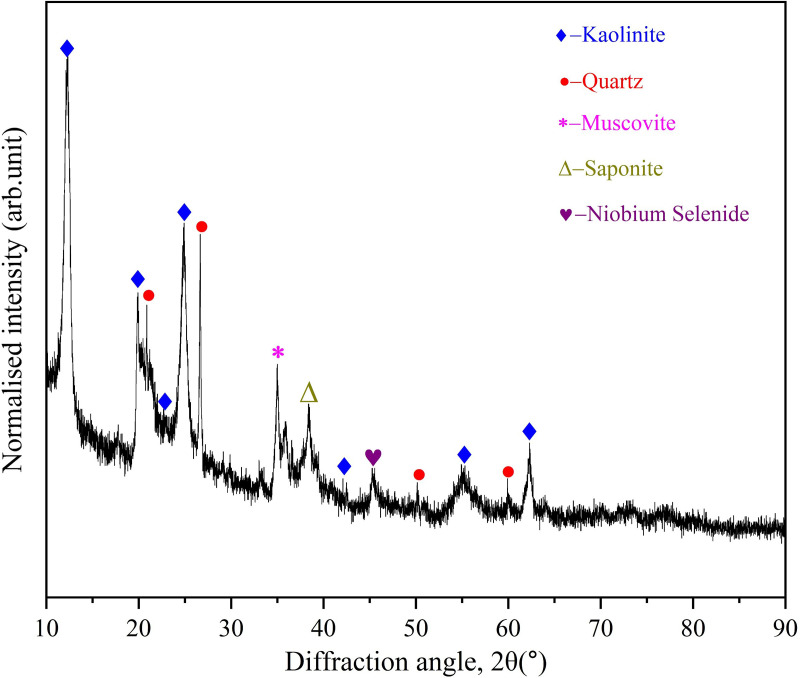
X ray diffraction (XRD) pattern of soil samples.

The X-ray diffraction (XRD) pattern of the treated soil samples is shown in Fig 2. Phase identification through the XRD standard pattern database reveals that the mineral phases in the soil samples are primarily kaolinite (Al_2_O_3_∙2SiO_2_ ∙ 2H_2_O) and quartz (SiO_2_), which is consistent with the high Si and Al content observed in the major element composition analysis.

### 2.2. Experimental apparatus

#### 2.2.1. Column leaching apparatus.

**Fig 3:**
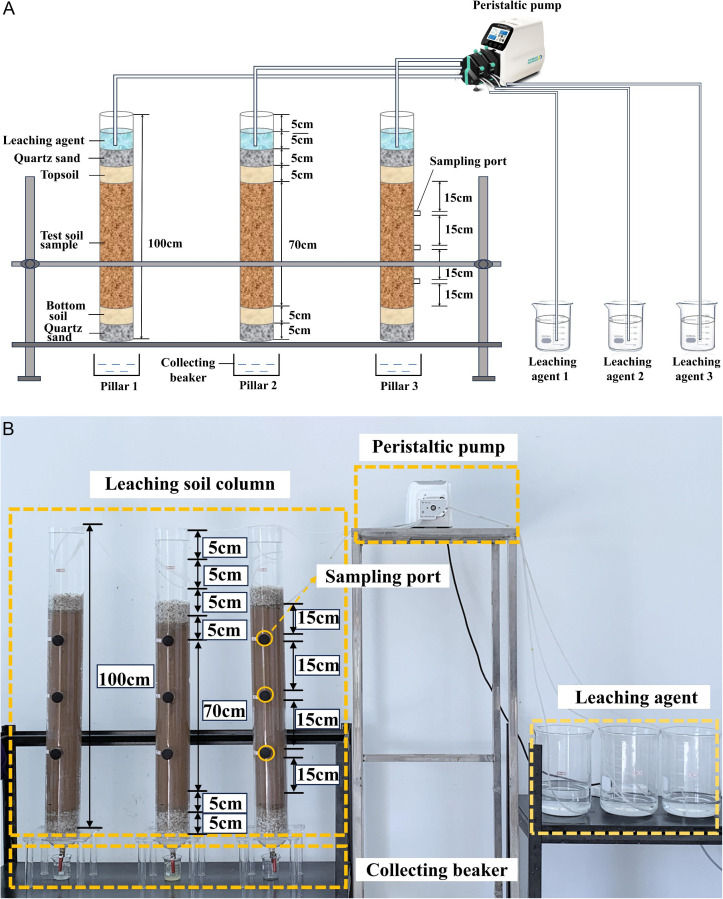
Schematic of the column leaching apparatus. (a) Schematic diagram of the column leaching apparatus. (b) Photo of the column leaching apparatus.

A self-made column leaching apparatus was used for conducting indoor simulated leaching experiments, as shown in Fig 3. The transparent PVC pipe used in the experiment has a height of 100 cm, an outer diameter of 110 mm, and an inner diameter of 100 mm. The column is pre-configured with three sampling holes, with a distance of 15 cm between the holes, and the first hole is 35 cm from the top of the column. The soil column is divided into six parts, from top to bottom: 0–10 cm from the top of the column is reserved for the leaching agent, 10–15 cm is the top quartz sand section, 15–20 cm is the topsoil section, 20–90 cm is the ore-containing soil section, 90–95 cm is the bottomsoil section, 95–100 cm is the bottom quartz sand section.

#### 2.2.2. ICP-OES detection apparatus.

**Fig 4:**
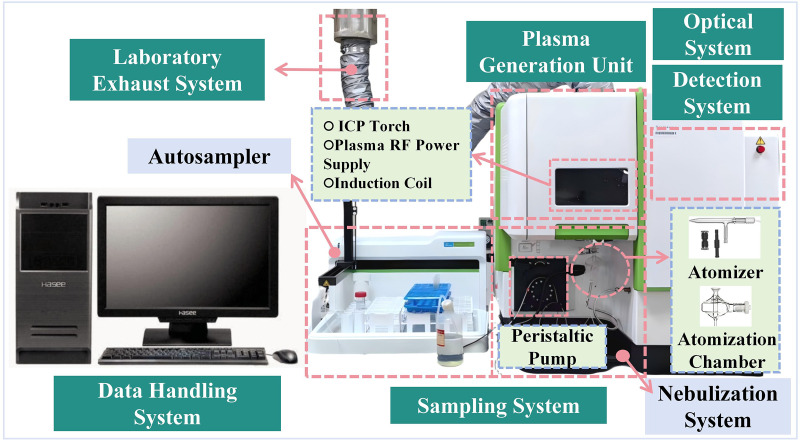
Schematic of the ICP-OES apparatus.

In this study, the subsequent tests for Zn concentration were performed using the ICP-OES Avio 200, manufactured by PerkinElmer Singapore Pte Ltd, as shown in Fig 4.

### 2.3. Experimental methods

#### 2.3.1. Column leaching experiment.

The physical parameters (water content and compaction) of the remolded soil samples in the experimental columns were strictly controlled according to the field conditions of the mine. The soil columns were constructed using a layered filling method, with each 20 cm layer compacted. After filling, initial soil samples were immediately collected from the sampling port at the mid-height of the column (specifically, 50 cm below the upper boundary of the top quartz sand layer and 60 cm from the column top) to obtain background values before the leaching process. In subsequent column leaching experiments, samples were consistently taken from the mid-column sampling port shown in Fig 3. Each sampling was performed in triplicate, and independent measurements were averaged.

This experiment uses the control variable method, designed to simulate leaching tests with different types and concentrations of leaching agents, based on the actual leachate injection conditions of ion-type rare earth in-situ leaching mining (the leaching agents typically include (NH_4_)_2_SO_4_, MgSO_4_, Al_2_(SO_4_)_3_, with concentrations ranging from 1% to 8%, and pH values between 2 and 5). To realistically simulate the leaching effect of the mine, the pH of the leaching agent was set to 2, with an injection rate of 2 ml/min. Detailed experimental conditions are shown in Table 2. The experiment was divided into two cycles, each lasting 7 days. The first cycle consisted of simulated leaching tests with different leaching agents ((NH_4_)_2_SO_4_, MgSO_4_, Al_2_(SO_4_)_3_), while the second cycle focused on simulated leaching tests with different concentrations of Al_2_(SO_4_)_3_ (1%, 3%, 5%, and 7%). During the leaching period (Days 1–7), leachate was collected daily from the liquid beaker, and the Zn concentration in the leachate was promptly measured. Daily samples were taken from the center sampling holes of the soil columns after leaching. The samples were then dried, ground, sieved through a 0.075 mm geotechnical sieve, and prepared for subsequent analysis.

**Table 2:** Design of different leaching conditions.

Column number	Type and concentration of lixiviant	pH	Leaching intensity (ml/min)
A1	3%(NH_4_)_2_SO_4_	2	2
A2	3%Al_2_(SO_4_)_3_	2	2
A3	3%MgSO_4_	2	2
B1	1%Al_2_(SO_4_)_3_	2	2
B2	5%Al_2_(SO_4_)_3_	2	2
B3	7%Al_2_(SO_4_)_3_	2	2

#### 2.3.2. Total Zn digestion test.

Metal elements in soil are typically bound within the lattice structure of minerals or compounds, making direct detection difficult to obtain accurate results. Therefore, soil digestion methods are employed to break the mineral lattice structure, converting Zn from its stable bound state to a soluble form [15,16]. This study employed a four-acid digestion system of HCl - HF – HClO_4_ - HNO_3_ to perform total Zn extraction from soil samples. The Zn content in the solution was then measured using ICP-OES, as shown in Fig 4.

#### 2.3.3. Continuous extraction experiment of Zn chemical forms.

The modified BCR continuous extraction method was used to determine the content and proportion of various Zn forms in the soil samples [17]. According to the BCR continuous extraction method, Zn in soil samples can be classified into four forms: acid-extractable (F_1_), reducible (F_2_), oxidizable (F_3_), and residual (F_4_). The specific steps of the BCR continuous extraction method are shown in S1 Table.

#### 2.3.4. Zn environmental risk assessment method.

According to the BCR sequential extraction method, the environmental risk assessment index (RAC) is introduced to evaluate the potential environmental risks associated with Zn, as shown in Equation (1).


$$\text{RAC}={\text{F}}_{\text{1}}/\left({\text{F}}_{\text{1}}+{\text{F}}_{\text{2}}+{\text{F}}_{\text{3}}+{\text{F}}_{\text{4}}\right)\times \text{100}\%$$ (1)

In the equation, the RAC value represents the environmental risk assessment index, while F_1_, F_2_, F_3_ and F4 represent the contents of the acid-extractable, reducible, oxidizable, and residual fractions in the BCR SEP, respectively [18]. According to the environmental risk assessment guidelines, when the RAC value of Zn in soil is below 1%, it is considered to pose no risk to the ecological environment, 1% − 10% is classified as low risk, 11% − 30% as medium risk, and 31% − 50% as high risk [19].

## 3. Results and discussion

### 3.1. Variation of Zn concentration in leachate under different leaching conditions

#### 3.1.1. Variation of Zn concentration in leachate under different leaching agents.

**Fig 5:**
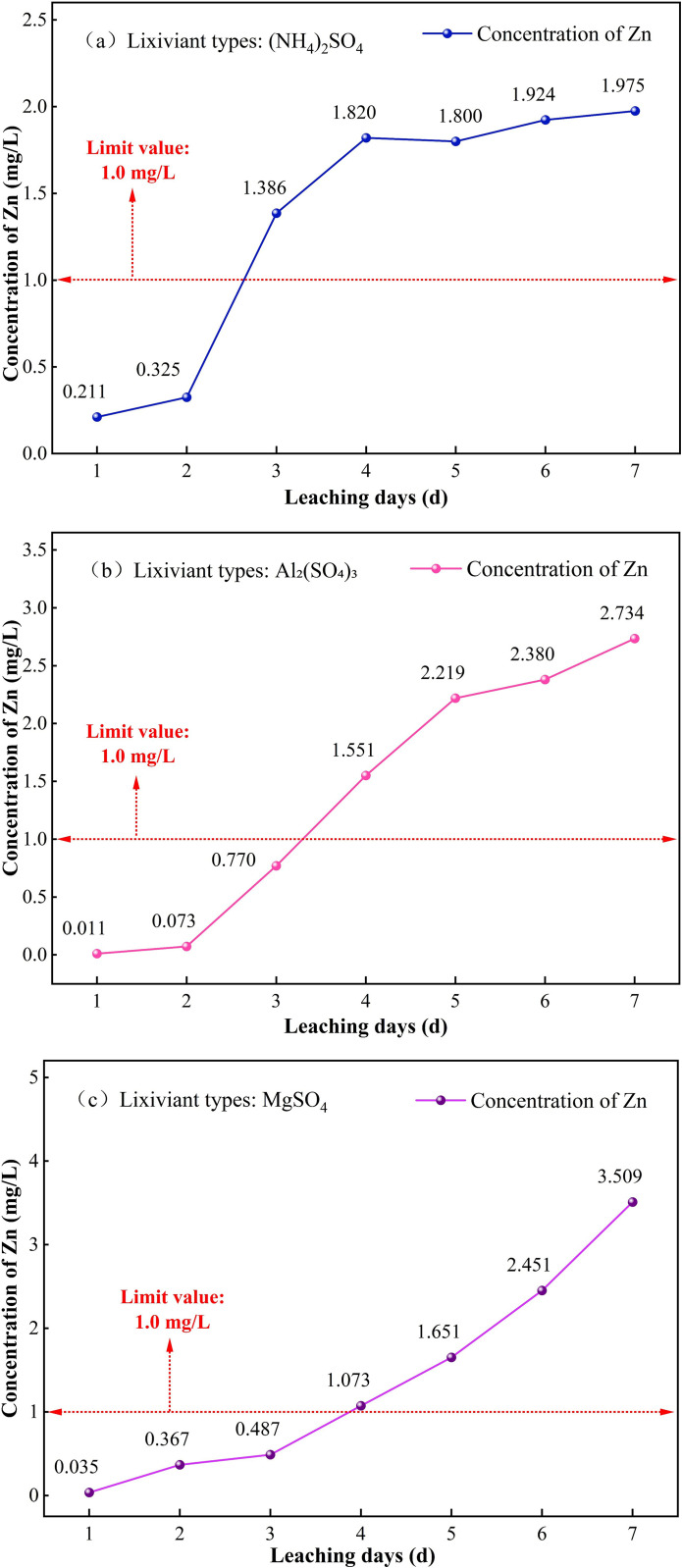
Variation of Zn concentration in leachate under different lixiviants (c = 3%). (a) (NH_4_)_2_SO_4_, (b) Al_2_(SO_4_)_3_, (c) MgSO_4_.

The variation of Zn concentration in the leachate under different leaching agents is shown in Fig 5. As shown in Fig 5(a), under the leaching of 3% (NH_4_)_2_SO_4_, the Zn concentration in the leachate exhibits a rapid increase in the early leaching stage (1–3 d). The Zn concentration in the leachate increased sharply from 0.2112 mg/L on day 1 to 1.386 mg/L on day 3, a 6.5-fold increase. In the middle and late stages of leaching (3–7 d), a slow increase trend was observed. It is believed that the rapid release of Zn in the early stage of leaching is due to the strong acidification effect of (NH_4_)_2_SO_4_, which causes the rapid dissolution of acid-soluble Zn in the soil [20]. At the same time, SO_4_^2-^ forms soluble complexes with Zn^2+^, further enhancing the mobility of Zn. In the middle and late stages of leaching, the active Zn in the soil gradually depletes, while the residual Zn is difficult to dissolve further due to its stable structure. Additionally, the NH_4_^+^ continuously supplied by (NH_4_)_2_SO_4_ may compete with Zn^2+^ for adsorption sites, inhibiting further desorption. This results in the Zn concentration in the leachate stabilizing in the middle and late stages of leaching.

As shown in Fig 5(b), under the leaching action of Al_2_(SO_4_)_3_ at a concentration of 3%, the Zn concentration in the leachate increases slowly at first and then rapidly during the early leaching stage (1–3 d), from 0.0107 mg/L on day 1 to a sudden increase of 0.7698 mg/L on day 3. During the middle and late leaching stages (3–7 d), the Zn concentration continues to increase at a rapid rate initially, then at a slower rate, with a significant decrease in the rate of increase in the later stage. The increase in Zn concentration in the leachate during the early leaching stage is primarily due to the hydrolysis of Al_2_(SO_4_)_3_, which generates a large amount of H^+^, maintaining a low pH environment, thereby facilitating the rapid dissolution of acid-soluble Zn in the soil. The continued release of Zn during the middle and late leaching stages may be attributed to the presence of a large amount of Al^3+^ in the leachate, which exchanges with Zn^2+^ adsorbed on soil colloids through ion exchange, disrupting the interlayer structure of clay minerals (such as kaolinite and montmorillonite), converting residual Zn into a more active form, which is then released into the leachate under further action of the leaching agent [21]. The decrease in the rate of increase in the later leaching stage may be due to the near depletion of the activatable Zn in the soil, with the dissolution-precipitation equilibrium tending to stabilize.

As observed in Fig 5(c), during the leaching process with 3% MgSO_4_ concentration, the Zn concentration in the leachate increases slowly in the early leaching stage (1–3 d). In the mid-to-late leaching stage (3–7 d), the concentration increased exponentially, surging from 0.4812 mg/L on day 3 to 3.509 mg/L on day 7, a rise of approximately 6.3 times. It is believed that in the early leaching stage, Mg^2+^ rapidly displaced the Zn^2+^ adsorbed on soil colloids through ion exchange, significantly enhancing the mobility of Zn. The rapid increase in Zn concentration in the leachate during the mid-to-late leaching stage may be due to continuous leachate injection, which enhances the acidifying effect of MgSO_4_, promoting the dissolution of weakly acidic extractable Zn. Simultaneously, a large amount of Mg^2+^ exchanged ions with the surface of soil particles, altering the soil colloidal structure and thereby desorbing a significant amount of Zn^2+^ from the soil particle surface into the leachate [22].

The peak concentrations of Zn in the leachate over the entire leaching period under the influence of three leaching agents were ranked as MgSO_4_ > Al_2_(SO_4_)_3_> (NH_4_)_2_SO_4_, all of which significantly exceeded the limit of 1.0 mg/L specified in the Chinese “Surface Water Environmental Quality Standard” (GB3838–2002). Among these, the peak concentration of Zn in the leachate under the MgSO_4_ leaching system was 3.509 mg/L, which was significantly higher than the peak concentrations in the (NH_4_)_2_SO_4_ and Al_2_(SO_4_)_3_ leaching systems, exceeding the limit by approximately 2.5 times. This indicates that the three leaching agents have a significant difference in their effect on the activation and release of Zn in the soil. The leachate under the MgSO_4_ leaching system may diffuse into surrounding water bodies, potentially increasing the bioavailability of Zn in aquatic ecosystems, which could subsequently lead to ecological toxicity effects through the food chain [23].

In the in-situ leaching process of ionic rare earth ores, the mid-to-late stages of the leaching process are critical for pollution control. Water quality monitoring should be strengthened, especially during the leachate discharge stage. Regular monitoring of Zn concentrations in the leachate should be conducted, and appropriate purification measures (e.g., chemical precipitation, adsorption, etc.) should be implemented to ensure that water bodies are not polluted.

#### 3.1.2. Variation of Zn concentration in leachate under different leaching agent concentrations.

**Fig 6:**
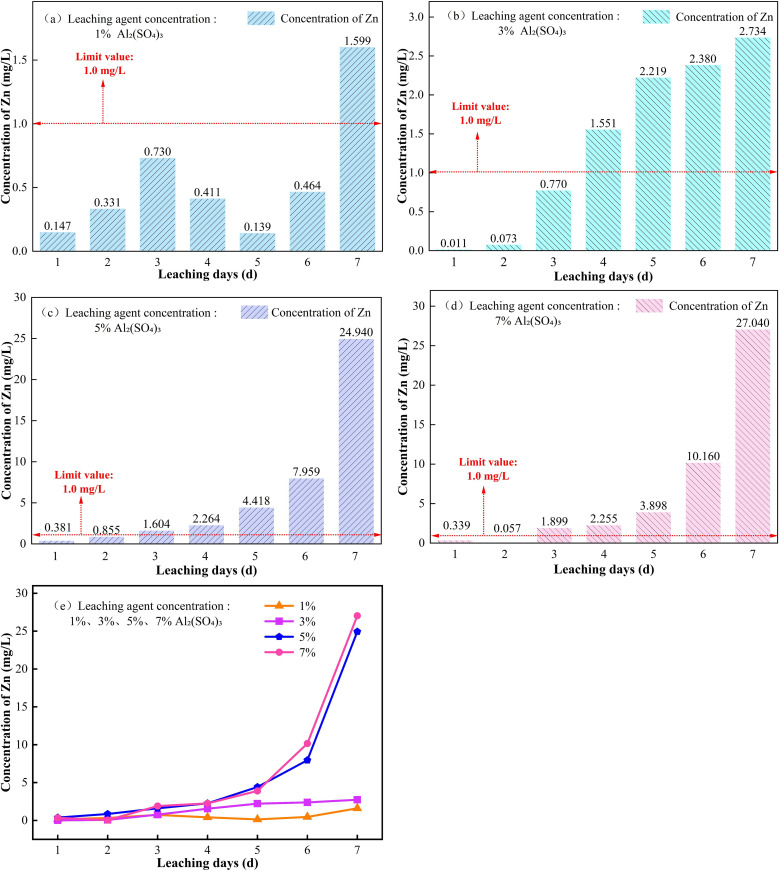
Variation of Zn concentration in leachate with leaching time under different Al_2_(SO_4_)_3_ concentrations. (a) 1%, (b) 3%, (c) 5%, (d) 7%, (e) 1, 3, 5, 7%.

The variation of Zn concentration in the leachate with leaching time under different Al_2_(SO_4_)_3_ concentrations is shown in Fig 6. As shown in Fig 6(a), under the leaching effect of 1% Al_2_(SO_4_)_3_, the Zn concentration in the leachate increased from 0.1469 mg/L to 0.7299 mg/L during the early stage of leaching (0–3 d), approximately a four-fold increase. During the mid-stage of leaching (3–5 d), it rapidly decreased to 0.1394 mg/L, a reduction of 80.9%. During the late stage of leaching (5–7 d), it surged from 0.1394 mg/L to 1.599 mg/L. It is believed that the rapid increase of Zn concentration in the leachate during the early stage of leaching is due to the strong acidity produced by the hydrolysis of Al^3+^, which can quickly dissolve Zn in weakly acidic extractable forms. Additionally, Al^3+^ facilitates the rapid release of Zn^2+^ through ion exchange, displacing colloid-adsorbed Zn^2+^.The sharp decrease of Zn concentration in the leachate during the mid-stage of leaching may be attributed to the adsorption of Zn^2+^ by Al(OH)_3_ colloids generated from Al^3+^ hydrolysis, as well as the inhibition of Zn^2+^ dissolution by ZnSO_4_ micro-precipitates. The continued increase in the late stage of leaching may be due to the continued erosion of clay minerals by Al^3+^, releasing residual Zn, and the dissolution of ZnSO_4_ precipitates formed in the early stage, resulting in secondary Zn release [24].

As observed in Fig 6(c), under the leaching effect of 5% Al_2_(SO_4_)_3_, the Zn concentration in the leachate increases slowly during the early and middle stages of leaching, and then increases exponentially in the later stages. It is analyzed that in the early stage of leaching, weakly acid-extractable Zn is mainly dissolved by the strong acidity generated by hydrolysis, and Al^3+^ releases reducible Zn through ion exchange. In the middle and later stages of leaching, on one hand, the higher concentration of Al^3+^ erodes the clay mineral lattice, activating the release of a large amount of residual Zn; on the other hand, SO_4_^2-^ forms highly stable complexes with Zn^2+^, inhibiting the precipitation of Zn(OH)_2_, resulting in an exponential increase in Zn concentration in the leachate.

As shown in Fig 6(d), under the leaching effect of 7% Al_2_(SO_4_)_3_, the Zn concentration in the leachate first decreases and then increases during the early stage of leaching, and in the middle and later stages, it shows an exponential increase, similar to the 5% Al_2_(SO_4_)_3_. It is analyzed that in the early stage of leaching, 7% Al_2_(SO_4_)_3_ hydrolyzes to generate a large amount of Al(OH)_3_ colloid, which adsorbs some Zn^2+^, inhibiting the activation and release of Zn. In the middle and later stages of leaching, due to continuous injection of the solution, the Al^3+^ content in the ore body increases, enhancing the erosion effect of Al^3+^ on the interlayer structure of clay minerals, and activating the release of residual Zn into the leachate, leading to an exponential increase in Zn concentration in the leachate.

Analysis of Fig 6(e) shows that the peak Zn concentration in the leachate of Al_2_(SO_4_)_3_ at high concentrations (5%, 7%) is significantly higher than that at low concentrations (1%, 3%) of Al_2_(SO_4_)_3_. The difference is believed to be caused by the greater disruption of the interlayer structure of clay minerals by Al_2_(SO_4_)_3_ in the high-concentration system, and the acidic environment produced that can effectively dissolve weakly acidic extractable Zn [25]. The peak Zn concentrations in the leachate after leaching with Al_2_(SO_4_)_3_ at all four concentrations significantly exceed the limit of 1.0 mg/L set by the Chinese “Surface Water Environmental Quality Standards” (GB3838–2002), and all occur in the later stages of the leaching process. At this point, the Zn release rate is the fastest, representing the critical stage for pollution control. When using Al_2_(SO_4_)_3_ for leaching, enhanced monitoring of water quality in the later stages of leaching is recommended.

### 3.2. Changes in Zn content in soil under different leaching conditions

#### 3.2.1. Changes in Zn content in soil under the action of different leaching agents.

**Fig 7:**
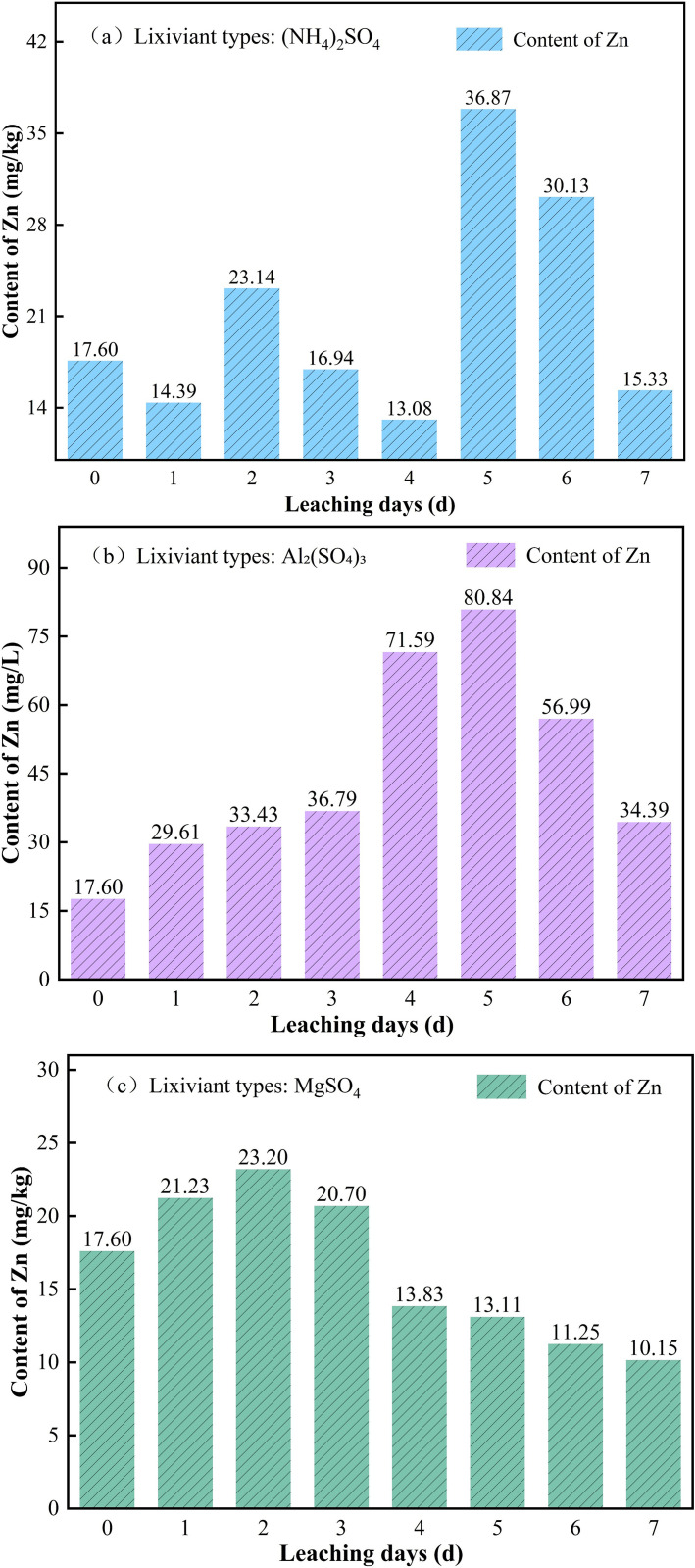
Changes in Zn concentration in soil under different leaching agents (c = 3%). (a) (NH_4_)_2_SO_4_, (b) Al_2_(SO_4_)_3_, (c) MgSO_4_.

The changes in Zn concentration in soil under different leaching agents are shown in Fig 7. As shown in Fig 7(a), under the leaching effect of (NH_4_)_2_SO_4_ at a concentration of 3%, the Zn content in soil initially decreased and then increased in the early leaching stage (0–2 d). In the mid-leaching stage (2–5 d), the Zn content showed a gradual decrease followed by a rapid increase, dropping sharply from 23.14 mg/kg on day 2 to 13.08 mg/kg on day 4, and then surging to 36.87 mg/kg on day 5, an increase of 181.8%. In the late leaching stage (5–7 d), a rapid decline in Zn content was observed. The reason for the initial decrease followed by an increase in the early leaching stage is that NH_4_^+^ exchanged some Zn^2+^ into the solution through ion exchange, and then SO_4_^2-^ formed soluble complexes with Zn^2+^, inhibiting re-adsorption, leading to the increase in soil Zn content [26]. The reason for the second occurrence of a decrease followed by an increase in the mid-leaching stage may be that the increase in Zn^2+^ concentration in the leachate triggered the ion effect, inhibiting the release of Zn, followed by continuous addition of the leaching solution, where the acidification effect of (NH_4_)_2_SO_4_ was enhanced, activating the stable Zn in the soil and promoting its release. The rapid decrease in soil Zn content in the late leaching stage is due to the majority of the Zn in the active state being displaced into the leachate, with the available Zn in the soil nearly depleted [27].

As shown in Fig 7(b), under the leaching effect of 3% Al_2_(SO_4_)_3_, the Zn content in the soil exhibited a rapid increase in the early leaching stage (0–2 d). The Zn content in the soil showed a slow then rapid increase in the mid-leaching stage (2–5 d), surging from 33.43 mg/kg on the 2nd day to 80.84 mg/kg on the 5th day, an increase of 142%, followed by a rapid decrease in the late leaching stage (5–7 d). It is analyzed that in the early leaching stage, Al_2_(SO_4_)_3_ dissolves Zn in its active state through strong acidity, and a large amount of Al^3+^ exchanges with Zn in the reducible state, temporarily converting it to the active state, resulting in an increase in soil Zn content. In the mid-leaching stage, due to the continuous injection of solution, the accumulated Al^3+^ severely damages the clay mineral structure, converting Zn in the residual state into the active state, while the coordination effect of SO_4_^2-^ inhibits the dissolution of Zn^2+^, leading to a continuous and rapid increase in soil Zn content [28]. The rapid decrease in the late leaching stage may be due to the continuous injection of solution enhancing the acidic effect of Al_2_(SO_4_)_3_, causing Zn in the active state in the soil to dissolve and release into the leachate. Meanwhile, the adsorption capacity of Al(OH)_3_ colloid decreases after aging, leading to the re-desorption of Zn^2+^ into the leachate.

As shown in Fig 7(c), under the leaching effect of 3% MgSO_4_, the Zn content in the soil exhibited a rapid increase in the early leaching stage (0–2 d), rising from 17.60 mg/kg to 23.20 mg/kg. The Zn content in the soil showed a decreasing trend in the mid-late leaching stage (2–7 d), continuously decreasing from 23.20 mg/kg on day 2 to 13.11 mg/kg on day 7, a decrease of 56.3%. The rapid increase in soil Zn content in the early leaching stage may be due to Mg^2+^ exchanging with Zn^2+^ adsorbed on soil colloids, while SO_4_^2-^ forms soluble complexes with Zn^2+^, leading to a temporary accumulation of Zn in the active state in the soil [29]. The continued decrease in the mid-late leaching stage may be due to, on one hand, Mg^2+^ exchanging with the surface of soil particles, desorbing Zn^2+^ from the surface into the leachate. On the other hand, the increased Zn^2+^ concentration in the leachate induces the common ion effect, causing the continuous release of Zn in the active state from the soil into the leachate [30].

As shown in Fig 7, during the entire leaching period using Al_2_(SO_4_)_3_, the peak Zn concentration in the soil was 80.84 mg/kg, which was significantly higher than that of the other two leaching agents, approaching the high-risk Zn concentration value specified in the “Classification of Heavy Metal Pollution in Agricultural Product Production Area Soils” (DB35/T 859–2016) of Fujian Province, China (Zn safe value: 20 mg/kg; limit value: 60 mg/kg; high-risk value: 90 mg/kg). This indicates that when Al_2_(SO_4_)_3_ is used for the leaching operation, a significant amount of Zn in its active form remains in the ore body during the later stages of the leaching process.

As shown in Fig 5 and 7, the peak Zn content in soil under MgSO_4_ leaching was 23.20 mg/kg, which was lower than that observed with the other two leaching agents; however, the peak Zn concentration in the leachate under MgSO_4_ leaching was significantly higher than that of the other two agents. This phenomenon is attributed to the fact that Mg^2+^ and Zn^2+^ share the same valence; the hydrolysis of MgSO_4_ produces a large amount of divalent Mg^2+^, which disrupts the adsorption/desorption equilibrium of Zn^2+^ on the soil surface, thereby promoting the desorption of Zn^2+^ from soil particles into the leachate.

For areas of soil in ion-adsorption rare earth mining regions with elevated Zn levels after leaching, post-treatment should be implemented in combination with soil remediation techniques, such as the application of soil stabilizers or adsorbents (e.g., bentonite, diatomite), to reduce the bioavailability of Zn in the soil and thereby mitigate its ecological risks.

#### 3.2.2. Changes in Zn content in soil under different concentrations of leaching reagents.

**Fig 8:**
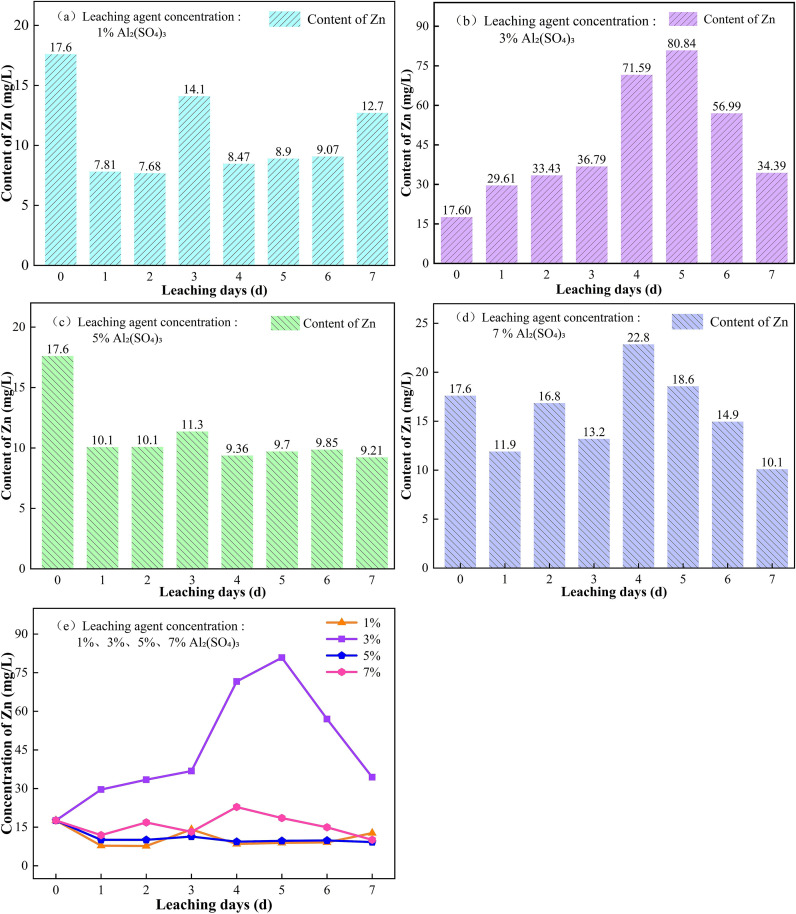
Changes in Zn content in soil under the leaching effect of different concentrations of Al_2_(SO_4_)_3_. (a) 1%, (b) 3%, (c) 5%, (d) 7%, (e) 1, 3, 5, 7%.

The changes in Zn content in soil under different concentrations of Al_2_(SO_4_)_3_ are shown in Fig 8. As shown in Fig 8(a), under the leaching effect of 1% Al_2_(SO_4_)_3_, the Zn content in the soil rapidly decreases in the early stage of leaching (0–2 d), dropping sharply from 17.60 mg/kg to 7.68 mg/kg. In the middle stage of leaching (2–5 d), the Zn content first increases and then decreases, rising from 7.68 mg/kg to 14.1 mg/kg, and then decreasing to 8.47 mg/kg. In the late stage of leaching (5–7 d), the Zn content in the soil shows an increasing trend. It is believed that in the early stage of leaching, the acidic conditions generated by the hydrolysis of Al^3+^ cause the Zn in the acid-extractable state within the ore body to dissolve into the leachate, resulting in a significant decrease in Zn content. During the middle stage of leaching, due to the lower concentration of Al_2_(SO_4_)_3_, the Al^3+^ provided is limited, and its ability to ion-exchange and release Zn is restricted. The activated Zn briefly accumulates in the soil, and with continued leachate injection, the Al^3+^ concentration increases, leading to further dissolution of Zn in its active state, causing the Zn content to decrease again [31]. In the late stage of leaching, the accumulation of Al^3+^ in the soil continuously damages the interlayer structure of clay minerals, leading to the activation and release of Zn in the residual state. Additionally, the aging of Al(OH)_3_ colloids results in the saturation of adsorption sites, causing the release of Zn from the ore body into the leachate and a subsequent decrease in Zn content in the soil.

In Fig 8(c), it can be observed that under the leaching effect of Al_2_(SO_4_)_3_ at a concentration of 5%, the soil Zn content significantly decreases in the early leaching period (0–2 d). In the middle and later stages of leaching (2–7 d), the soil Zn content exhibits fluctuating changes, specifically increasing first, then decreasing, followed by a slow recovery and subsequent decline. The significant decrease in soil Zn content in the early leaching period is caused by the dissolution of acid-extractable Zn. This dynamic change in the middle and later stages of leaching reflects the activation-dissolution-adsorption process of different forms of Zn during the leaching process.

In Fig 8(d), it can be seen that under the leaching effect of Al_2_(SO_4_)_3_ at a concentration of 7%, the fluctuation of soil Zn content becomes more pronounced throughout the entire leaching period. This is because the continuous injection of Al_2_(SO_4_)_3_ at a higher concentration provides a large amount of Al^3+^, which erodes and disrupts the interlayer structure of clay minerals, converting residual Zn to its active form. At the same time, Al(OH)_3_ colloids are easily formed, which adsorb free Zn^2+^ in the soil, leading to an increase in soil Zn. However, with the increase in injection time, the high concentration of Al_2_(SO_4_)_3_ can hydrolyze to create a more acidic environment, dissolving Zn in its active form and Zn adsorbed in colloids in the mineral body, leading to a decrease in soil Zn. The change in soil Zn content under high-concentration Al_2_(SO_4_)_3_ leaching reflects a more significant dynamic process of Zn activation-dissolution-adsorption during leaching [32].

From the comparative analysis of Fig 8(e), it is evident that in the Al_2_(SO_4_)_3_ leaching system, Al^3+^ primarily releases active Zn through acid dissolution and ion exchange in the early leaching period. In the middle leaching period, colloidal adsorption and desorption, along with the disruption of mineral structure and the dissolution of active Zn, lead to fluctuations in Zn content. However, in the later leaching period, the strongly acidic environment generated by continuous injection, colloidal aging, and the common ion effect greatly promote the release of Zn from the soil into the leachate [33]. As shown in Fig 8(e), the Zn content in the soil after leaching with a 3% Al_2_(SO_4_)_3_ solution is higher than that of the other three concentrations and approaches the high-risk value of Zn content in soil as defined by the “Classification of Soil Heavy Metal Pollution in Agricultural Product Production Areas” (DB35/T 859–2016) in Fujian Province, China. This indicates that during the leaching process with a 3% Al_2_(SO_4_)_3_ solution, the activation efficiency of Zn in the soil is significantly higher than its dissolution efficiency, resulting in a large amount of Zn remaining in an active state within the ore body. When selecting Al_2_(SO_4_)_3_ as a leaching agent, its optimal usage conditions should be determined through experiments, optimizing the concentration, dosage, and duration of Al_2_(SO_4_)_3_ to control the activation degree of Zn and avoid excessive activation of Zn due to overly high concentrations.

### 3.3. Chemical speciation analysis of Zn in soil under different leaching conditions

#### 3.3.1. Chemical speciation analysis of Zn in soil under different leaching agents.

**Fig 9:**
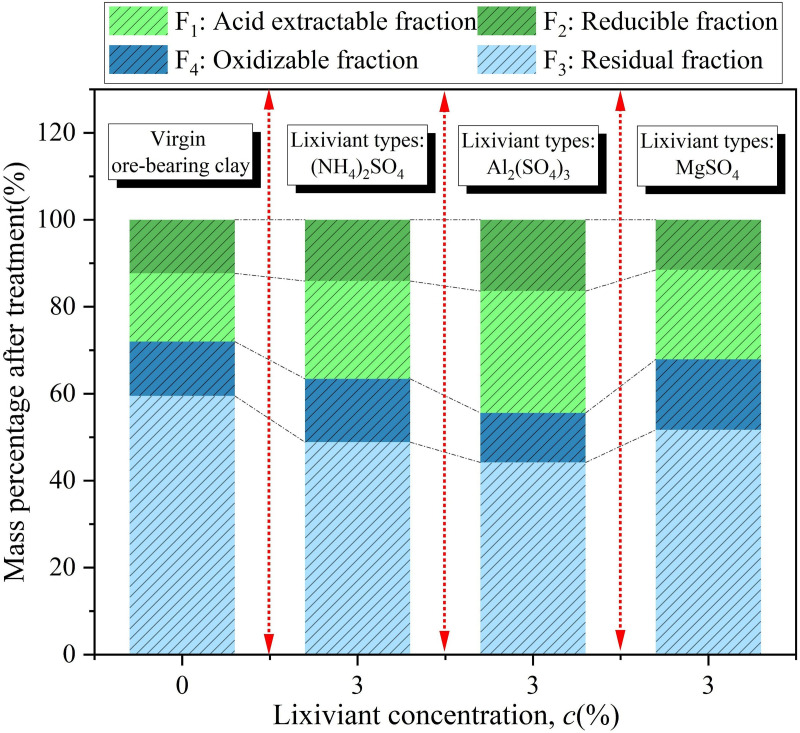
Proportions of different chemical forms of Zn in soil after the application of various lixiviants.

The proportions of different chemical forms of Zn in the soil after leaching with different leaching agents are shown in Fig 9. It can be observed from the figure that the proportions of different chemical forms of Zn in the original soil are as follows: F_1_ is 15.7%, F_2_ is 12.3%, F_3_ is 12.5%, and F_4_ is 59.5%, with a total of 40.5% in the bioavailable forms. This indicates that in the absence of leaching agents, Zn in the soil primarily exists in a residual form, with relatively less in bioavailable forms, limiting its mobility and bioavailability in the environment. It can also be observed from Fig 9 that after leaching with (NH_4_)_2_SO_4_, the proportion of F_1_ increases significantly to 22.5%, F_2_ rises to 14.1%, and F_3_ increases to 14.6%, with a total of 51.1% in bioavailable forms, while the residual form decreases to 48.9%. This indicates that (NH_4_)_2_SO_4_ effectively promotes the transformation of relatively stable Zn in the soil into bioavailable forms. (NH_4_)_2_SO_4_ primarily relies on the acidification effect of NH_4_^+^ to dissolve Zn in the soil that is in the acid-extractable form, enhancing the mobility of Zn in the surrounding soil [34,35].

After the leaching process with Al_2_(SO_4_)_3_, the proportion of Zn in F_1_ increased to 28%, F_2_ was 16.4%, and F3 was 11.4%. The total proportion of active Zn (F_1_, F_2_, F_3_) was 55.8%, while the proportion of residual Zn (F_4_) decreased to 44.2%. Compared to the leaching results with (NH_4_)_2_SO_4_, Al_2_(SO_4_)_3_ was more effective in increasing the proportion of active Zn, particularly in enhancing the proportion of acid-extractable Zn. Al₂(SO_4_)_3_ promotes the activation and release of Zn in the soil through its strong acidity and by disrupting the mineral structure, significantly enhancing the mobility and bioavailability of Zn in the soil. After the leaching process with MgSO_4_, the proportion of Zn in F_1_ was 20.6%, F_2_ was 11.5%, and F_3_ was 16.2%. The total proportion of active Zn (F_1_, F_2_, F_3_) was 48.3%, while the proportion of residual Zn was 51.7%. Compared to the original soil, the proportion of active Zn slightly increased, while the residual Zn still accounted for the majority. This indicates that MgSO_4_ had a relatively weak effect on altering the chemical forms of Zn in the soil, and its overall effect was less pronounced than that of (NH_4_)_2_SO_4_ and Al_2_(SO_4_)_3_. MgSO_4_ primarily works through ion exchange between Mg^2^⁺ and the soil particle surface, desorbing Zn^2+^ from the surface of the soil particles into the leachate [36,37].

**Fig 10:**
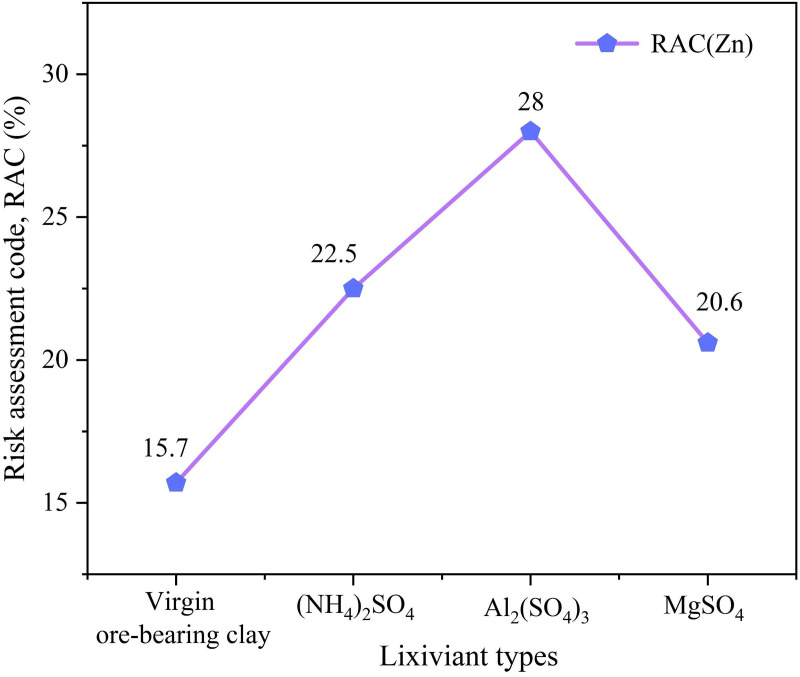
RAC values of Zn in soil after leaching with different leaching agents.

All three leaching agents increased the proportion of active Zn and decreased the proportion of residual Zn, indicating that they all disrupted the original balance of Zn chemical forms to some extent, promoting the conversion of Zn into more active forms and enhancing its activity in the soil environment. As shown in Fig 10, the RAC values of soil Zn treated with the three leaching agents ranged from 20% to 30%, indicating a moderate environmental risk level. Among them, the RAC value of soil Zn after leaching with Al₂(SO_4_)_3_ was the highest, indicating a greater potential ecological risk to the surrounding soil environment, making it unsuitable for mining operations in ecologically sensitive areas. On the other hand, the RAC value of the MgSO_4_ leaching system was the lowest, indicating a relatively lower environmental risk, making it suitable for low-intensity mining in ecologically sensitive areas. In practical engineering applications, it is necessary to comprehensively balance leaching efficiency and environmental risks, prioritizing the use of low-risk leaching agents to achieve a synergistic optimization of resource extraction and ecological sustainability [38].

#### 3.3.2. Chemical speciation of Zn in soil under different leaching agent concentrations.

**Fig 11:**
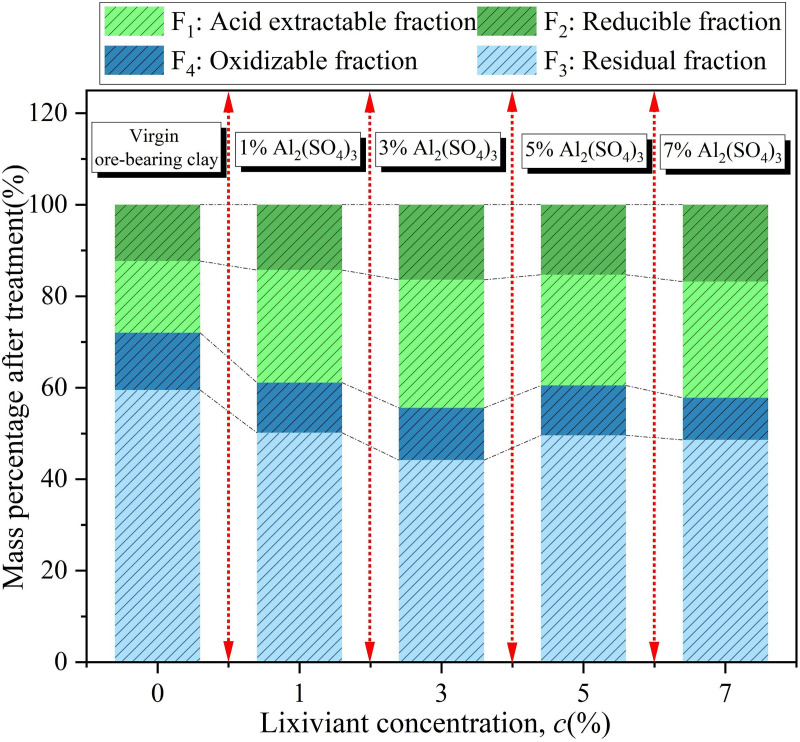
Proportion of different chemical forms of Zn in soil after treatment with different concentrations of Al_2_(SO_4_)_3_.

The chemical speciation of Zn in soil after leaching with different concentrations of Al_2_(SO_4_)_3_ is shown in Fig 11. As shown in the figure, under the leaching effect of 1% Al_2_(SO_4_)_3_, the proportion of Zn in F_1_ increased significantly to 24.6%, while F_2_ decreased to 11%, and F_3_ increased to 14.3%. The total proportion of the labile fraction accounted for 49.8%, while the residual fraction decreased to 50.2%. This indicates that the 1% concentration of Al_2_(SO_4_)_3_ can promote the transformation of some relatively stable Zn species in soil into labile forms, significantly altering the distribution of Zn chemical speciation, and enhancing the mobility and bioavailability of Zn in soil. When the Al_2_(SO_4_)_3_ concentration increased to 3%, the proportion of F_1_ further increased to 28%, F_2_ rose to 16.4%, and F_3_ decreased to 11.4%. The labile fraction accounted for 55.8%, while the residual fraction decreased to 44.2%. Compared to 1% Al_2_(SO_4_)_3_, 3% Al_2_(SO_4_)_3_ has a more significant effect in increasing the proportion of labile Zn.

Under the leaching of Al_2_(SO_4_)_3_ at a concentration of 5%, F_1_ accounted for 24.2%, F_2_ increased to 15.3%, and F_3_ decreased to 10.9%. The total proportion of active states was 50.4%, while the residue state (F_4_) accounted for 49.6%. The proportion of active states decreased compared to the 3% Al₂(SO_4_)_3_ treatment but was still higher than the proportion in the original soil and at the 1% concentration. This indicates that, compared to the 3% concentration of Al₂(SO_4_)_3_, the 5% concentration of Al₂(SO_4_)_3_ causes relatively less damage to the interlayer structure of the ore body. When the concentration of Al₂(SO_4_)_3_ increased to 7%, F_1_ accounted for 25.4%, F_2_ rose to 16.8%, and F_3_ decreased to 9.2%. The total proportion of active states was 51.4%, while the residue state (F_4_) accounted for 48.6%. At this point, the proportion of active states was similar to that observed at the 5% concentration of Al₂(SO_4_)_3_. This suggests that as the concentration increases, the effect of Al₂(SO_4_)_3_ on the proportion of active Zn gradually diminishes. Other factors, such as competition from other cations in the soil and shifts in chemical equilibrium, may restrict the transformation of Zn during the leaching process [28].

**Fig 12:**
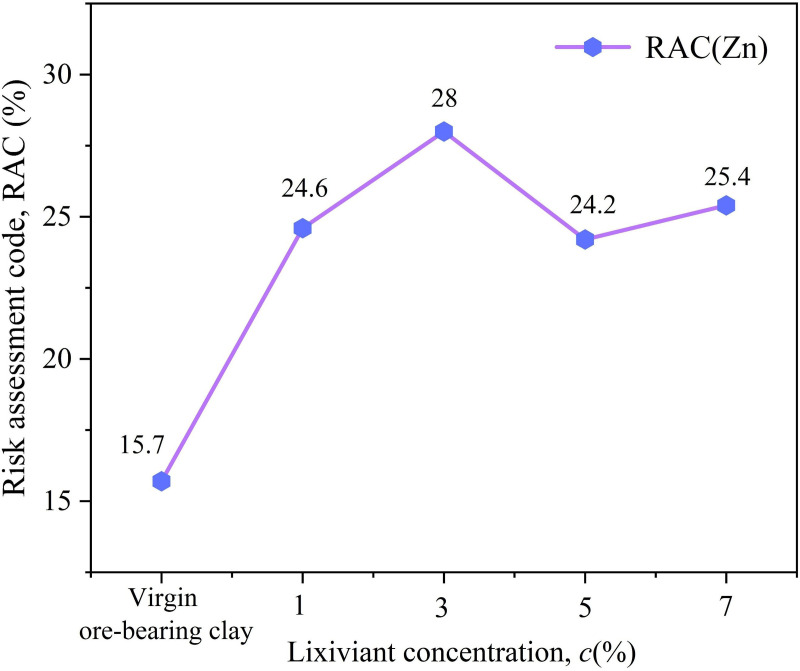
RAC values of Zn in soil after treatment with different concentrations of Al_2_(SO_4_)_3_.

The RAC values of Zn in the soil after treatment with different concentrations of Al₂(SO_4_)_3_ are shown in Fig 12. As seen in the figure, the RAC values of Zn in the soil after leaching with four different concentrations of Al₂(SO_4_)_3_ ranged from 20% to 30%, indicating a moderate environmental risk level. The RAC value was highest after leaching with 3% Al_2_(SO_4_)_3_. This indicates that the 3% concentration of Al₂(SO_4_)_3_ has the most significant promoting effect on the conversion of residue Zn to acid-extractable Zn. After leaching, a large amount of Zn remains in the acid-extractable state in the soil and has not been released into the leachate, posing a higher potential ecological risk [39]. When designing the concentration of Al_2_(SO_4_)_3_, it is advisable to avoid using a 3% concentration of Al_2_(SO_4_)_3_ in order to reduce the transformation of residual Zn in the soil to the acid-extractable form, thus ensuring the leaching efficiency of rare earth elements while controlling environmental risks within an acceptable range.

## 4. Conclusion

1) Significant differences exist in the activation and release patterns of Zn in soil under the influence of three leaching agents ((NH_4_)_2_SO_4_, MgSO_4_, Al_2_(SO_4_)_3_), with peak concentrations of Zn in the leachate following the order of MgSO_4_ > Al_2_(SO_4_)_3_> (NH_4_)_2_SO_4_, all of which significantly exceed the environmental limit value (Zn < 1.0 mg/L). The peak Zn content in the soil follows the order of Al_2_(SO_4_)_3_> (NH_4_)_2_SO_4_ > MgSO_4_. The later stages of the leaching process are key to pollution control. In the actual leaching process, it is crucial to strengthen the monitoring of water quality in the leachate discharge stages and adopt appropriate purification measures (such as chemical precipitation, adsorption methods, etc.) to ensure that water bodies are not polluted.
2) The peak Zn concentrations in the leachate under high-concentration systems (5%, 7%) of Al_2_(SO_4_)_3_ were significantly higher than those under low-concentration systems (1%, 3%) of Al_2_(SO_4_)_3_. The Zn content in the soil under 3% Al_2_(SO_4_)_3_ leaching was much higher than that under the other three concentrations (1%, 5%, 7%) of Al_2_(SO_4_)_3_, with a substantial amount of Zn remaining in the active state within the ore body after leaching at this concentration. When selecting Al_2_(SO_4_)_3_ as the leaching agent, the optimal usage conditions should be determined through experiments. Furthermore, the concentration, dosage, and duration of Al_2_(SO_4_)_3_ should be optimized to control the activation degree of Zn.
3) (NH_4_)_2_SO_4_ primarily promotes the activation and release of Zn through ion exchange between NH_4_^+^ and Zn^2+^ and its acidification effect. Al_2_(SO_4_)_3_ dominates the activation and release of Zn by providing a strongly acidic environment and dissolving the mineral lattice. MgSO4, in addition to ion exchange between Mg^2+^ and Zn^2+^, also alters the soil colloidal structure, thereby promoting the activation and release of Zn.
4) The promoting effect of the three leaching agents on the transformation of residual Zn in soil to the active state was ranked as Al_2_(SO_4_)_3_> (NH_4_)_2_SO_4_ > MgSO_4_, with the highest RAC value after Al_2_(SO_4_)_3_ leaching, indicating the greatest potential environmental risk. In contrast, the RAC value after MgSO4 leaching was the lowest, indicating relatively smaller potential environmental risk. In practical engineering applications, it is necessary to comprehensively balance leaching efficiency and environmental risks, prioritizing the use of low-risk leaching agents to achieve a synergistic optimization of resource extraction and ecological sustainability.
5) Compared to Al_2_(SO_4_)_3_ at concentrations of 1%, 5%, and 7%, 3% Al_2_(SO_4_)_3_ exhibited the most significant promoting effect on the transformation of residual Zn in soil to the acid-extractable state, with the highest RAC value and the greatest associated environmental risk. When designing the concentration of Al_2_(SO_4_)_3_, it may be considered to avoid using a 3% concentration of Al_2_(SO_4_)_3_ in order to reduce the conversion of residual Zn in the soil to the acid-extractable form, thus ensuring leaching efficiency while controlling environmental risks within an acceptable range.

## Supporting information

S1 Table Steps of the BCR continuous extraction method. (DOCX)
