# Supplementary material for: Study on the release pattern of Zn in soil of ionic rare earth mining areas under different leaching conditions
Source: PLoS One. 2025 Dec 15;20(12):e0338566. doi: 10.1371/journal.pone.0338566 (PMC12704852; doi:10.1371/journal.pone.0338566)
Supplement: S1 Table — (DOCX) [file pone.0338566.s001.docx]

**S1 Table Steps of the BCR continuous extraction method.**

| Steps | Form Types | Extractant | Extraction Time | Temperature |
| --- | --- | --- | --- | --- |
| 1 | F_1_ | 20 mL 0.11 mol/L HAC | 16 h | 25℃ |
| 2 | F_2_ | 20 mL 0.5 mol/L NH_2_OH∙HCl（pH=1.5^a^） | 16 h | 25℃ |
| 3 | F_3_ | 5 mL 30% H_2_O_2_ | 1h/1h | 25℃/85℃ |
|  |  | 5 mL 30% H_2_O_2_ | 1h | 85℃ |
|  |  | 25 mL 1 mol/L NH_4_Ac（pH=2^a^） | 16 h | 25℃ |
| 4 | F_4_ | 10 mL HF | 0.5h | 235℃ |
|  |  | 4 mL HClO_4_ | 0.5h | 265℃ |
|  |  | 10 mL HNO_3_ | 0.5h | 235℃ |
|  |  | 10 mL HNO_3_+15 mL H_2_O+1 mL H_2_O_2_ | 0.5 h | 235℃ |

Note: The pH value marked with "a" was adjusted using HNO_3_; 1h+1h means that the sample is digested at room temperature (25°C) for 1 hour, followed by further digestion in a water bath at 85°C for 1 hour.
